# Can you see me? Participant experience of accessing a weight management programme via group videoconference to overcome barriers to engagement

**DOI:** 10.1111/hex.13148

**Published:** 2020-10-22

**Authors:** Marion Cliffe, Enzo Di Battista, Simon Bishop

**Affiliations:** ^1^ Betsi Cadwaladr University Health Board Bangor Wales UK; ^2^ Aneurin Bevan University Health Board Newport Wales UK; ^3^ Bangor University Bangor Wales UK

**Keywords:** digital, obesity, rural health, self‐determination theory, videoconference, weight management

## Abstract

**Background:**

Engagement with conventional weight management group programmes is low.

**Objective:**

To understand participant experience of accessing an adapted programme via videoconference.

**Participants:**

Adults with obesity (BMI ≥ 35kg/m^2^), referred to an NHS Dietetics service in Wales, were offered a group videoconference weight management programme as an optional alternative to in‐person groups. Thirteen participants (mean age 48.5 ± 20.2 years, 8 female) recruited to two videoconference groups were interviewed.

**Study design:**

A Registered Dietitian delivered a behavioural programme using Skype for Business in 10 sessions over 6 months. Participants joined the groups from any Internet‐connected device with a webcam. Participant perspectives were audiorecorded in one‐to‐one, semi‐structured interviews. Interviews were transcribed verbatim and thematically analysed using self‐determination theory as a theoretical framework.

**Results:**

Ten themes were identified, three relating to service engagement and seven relating to behaviour change facilitation. Key themes in engagement included ‘reduced burden’, described as saving time and travel and ‘reduced threat’ as participants perceived joining a group from home as less daunting compared to attending in‐person. Despite reporting some initial technical difficulties with establishing video and audio connection, participants described beneficial peer support although not physically with other group members.

**Conclusion:**

Accessing a group weight management programme via videoconference may be the preferred option for some participants, overcoming some of the barriers to access to standard in‐person programmes, particularly in rural areas. Participants are able to experience peer support via videoconference. During the COVID‐19 pandemic, weight management programmes could utilize videoconference groups to continue to provide support.

## 1 BACKGROUND

Since 1975, worldwide obesity has nearly trebled, now affecting 650 million people,^1^ and in Wales 24% of adults live with obesity.^2^ Obesity is associated with reduced disability‐free‐life‐years and increased risk of co‐morbidities such as diabetes, heart disease, cancer and osteoarthritis.^3^ More recently, obesity has been associated with increased severity of coronavirus symptoms.^4^

Expert dietetic and behavioural counselling, as part of a structured programme with intensive follow‐up, can help people with obesity to achieve modest but clinically significant weight loss of 5kg or more.^5^ Evidence suggests that group‐based interventions may be more effective in facilitating weight change than individual interventions.^6^ The challenge, however, is that engagement with conventional, in‐person behavioural weight management programmes is known to be low.^7^ In a large multicentre trial of primary care referral to a group behavioural weight loss programme in England, only 6.5% of invited patients took up the offer.^8^ In Scotland, 34% of patients referred to a weight management service attended the first session.^5^

In Wales, approximately one in three people live in a rural area. Due to extended travel distances and comparatively poor public transport in rural areas, accessing services can be more time consuming, deterring some patients from utilizing health services.^9^ In a phenomenon described as ‘distance decay’ in rural residents, there is a decreasing rate of service use with increasing distance from services.^10^ Rural patients are less likely to have the opportunity to exercise choice in health care.^11^ Disparities in accessing care are especially relevant in weight management where participants need to access numerous sessions for support with health behaviour change.^12^

Harnessing the potential of digital technology to empower people to take greater control of their health and well‐being is of importance in Wales and internationally.^13^ Meta‐analyses of studies using eHealth interventions and mobile apps to promote weight loss have demonstrated significantly greater weight loss than controls.^14^, ^15^, ^16^ Evidence suggests that the addition of personalized or human interaction improves engagement with eHealth^14^, ^17^ and studies have shown that greater engagement is associated with greater weight loss.^18^, ^19^, ^20^, ^21^ A systematic review of a variety of group therapy and health education telehealth group programmes reported that groups delivered by videoconference are feasible and can potentially improve the accessibility of group interventions.^22^ Banbury et al^22^ concluded that videoconference group programmes in health care may be particularly useful for those who live in rural areas, have limited mobility, are socially isolated or fear meeting new people.

Recent studies have suggested that short‐term weight loss outcomes are at least as good as standard interventions when lifestyle programmes, including diet and exercise coaching, are delivered via videoconference.^3^, ^23^, ^24^, ^25^ Offering the option of accessing weight management programmes via videoconference could improve access to services and mitigate the travel and environmental costs of conventional in‐person services.^26^ Indeed, videoconferencing requires, at most, 7% of the total energy of an in‐person meeting.^27^ Participant acceptability, or satisfaction with, one‐to‐one videoconference weight management interventions, has been investigated in adults living in rural areas and in postpartum women.^12^, ^28^ However, to the authors’ knowledge, there are no qualitative studies reporting participants' experiences of accessing a group weight management programme via videoconference.

Medical Research Council guidance on evaluating complex interventions recommends that evaluations draw on existing theories on the mechanisms through which interventions bring about change.^29^ This study draws on the self‐determination theory (SDT) model of health behaviour change, which suggests that meeting the three psychological needs of autonomy, competence and relatedness can enable health behaviour change.^30^

Autonomy can be described as the psychological need to experience self‐direction and personal endorsement in the initiation and regulation of one's behaviour. The internalization of behaviour regulation is the process of taking in an idea, and transforming it into one's own.^31^ Autonomy satisfaction can be recognized in personal ownership of action.^32^ Competence refers to the need to be effective. In this context, competence describes a person's confidence in mastering healthy eating or skills to overcome challenges to changing eating behaviours. Promoting competence involves helping participants to experience mastery of change.^33^ Relatedness is the need to establish attachments with other people. The hallmarks of relatedness satisfaction are feeling socially connected and being actively engaged in both the giving and receiving of care to other people..^32^, ^33^ A concern regarding videoconference interventions is that, without authentic in‐person interaction, interpersonal connection might be diminished, groups may not fulfil relatedness needs, and consequently, this could jeopardize behaviour change initiation.^34^

The aim of this study was to qualitatively investigate participants’ perceptions of a group weight management programme, delivered via videoconference. Specifically, in order to understand:

- the acceptability of this digital approach for participants in a health setting.
- the impact of remote delivery on behaviour change facilitation, especially participants’ perceptions of relatedness or peer support.

## 2 METHODS

### 2.1 Participants

**Fig. 1:**
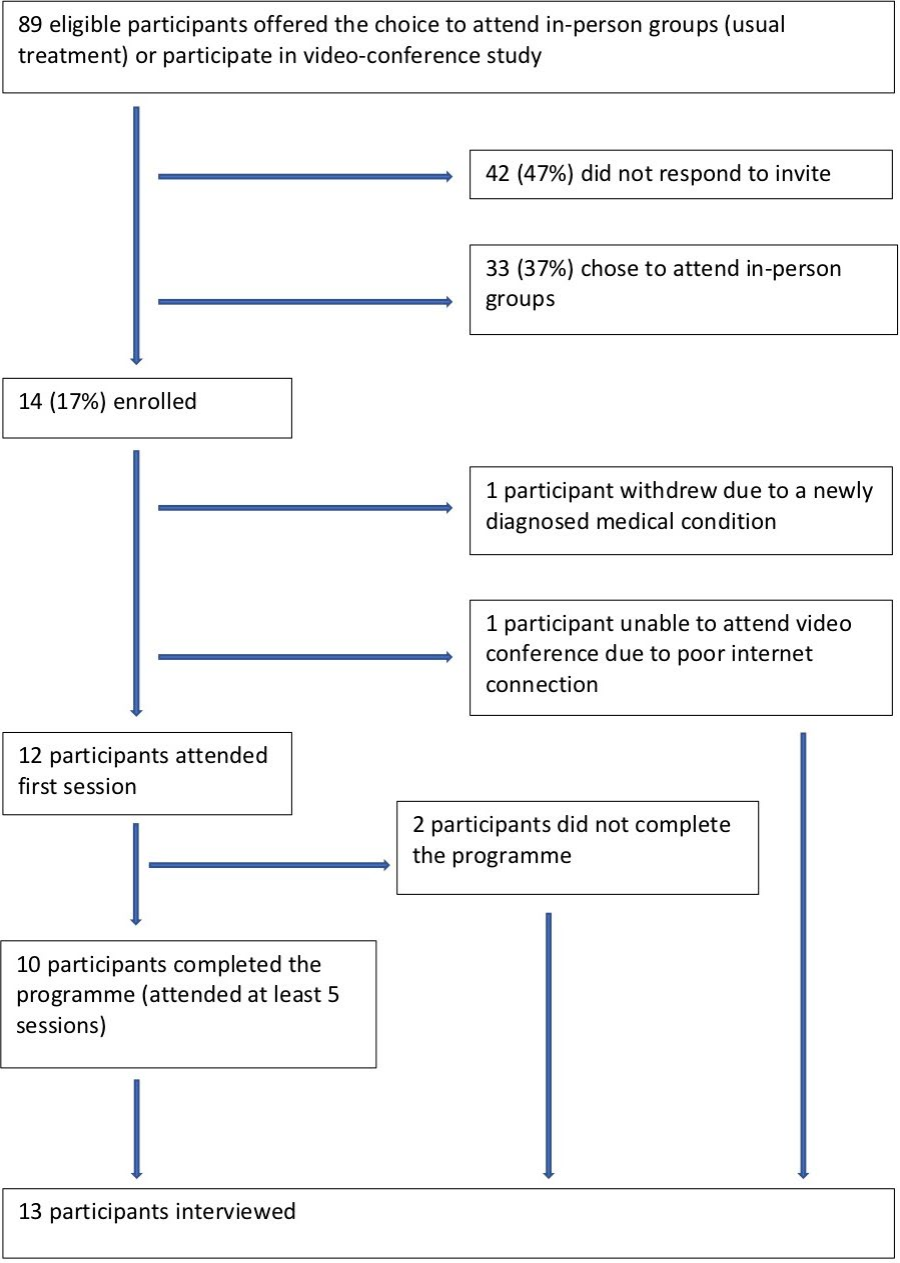
Participant flow diagram

Study ethical approval was granted by Wales Research Ethics Committee 5 on 26/11/2018. Participants were adults (≥18 years old) with BMI 35‐45kg/m^2^. Potential participants (n = 89) were referred by primary care, secondary care or self‐referral for weight management to an NHS Dietetics Service in Wales. For study inclusion, participants were required to be able to access an Internet‐connected device with camera and microphone in a private area of their choice, each week, at the time of the scheduled group. Exclusion criteria were pregnancy and known eating disorder disclosed at screening. Participants were offered the opportunity to enrol on the study intervention, a group videoconference programme, as an optional alternative to usual treatment. Only participants who chose to attend the videoconference intervention (n = 14) were enrolled on the study. Potential participants who did not respond to the invite or who chose to attend an in‐person programme were not interviewed. Participant flow is shown in Figure 1, and baseline characteristics of interviewed participants are shown in Table 1. Mean age of interviewed participants (n = 13, 5 male) was 48.5 ± 20.2 years, and mean BMI was 39.3 ± 2.9kg/m^2^.

**Table 1:** Socio‐demographic and medical characteristics of interviewed participants (n = 13)

		n = 13
Age (years)	18‐39	5
	40‐59	3
	≥60	5
Sex	Female	8
	Male	5
Ethnicity	White British	11
	White European	2
BMI (kg/m^2^)	Obesity class 2 (BMI 35‐39.99)	6
	Obesity class 3 (BMI 40‐44.99)	7
Co‐morbidities (participants with more than one co‐morbidity are counted more than once)	Osteoarthritis	6
	Depression/anxiety	5
	Type 2 diabetes/pre‐diabetes	3
	Polycystic ovary syndrome	3
	Hypertension	2
	Sleep apnoea	1
	Cardiovascular disease	1
	Other	1
Employment status	Full‐time	5
	Part‐time	2
	Retired	3
	Student	2
	Carer	1
Deprivation index (quintiles)^a^	1: most deprived	0
	2	1
	3	4
	4	6
	5: least deprived	2
Rurality^a^	Rural	7
	Urban	6
Distance to nearest venue (miles)	0‐9.9	7
	10‐19.9	1
	≥20	5

^a^ Welsh Index of Multiple Deprivation^53^

### 2.2 Intervention

The study intervention was adapted from a dietitian‐led weight management face‐to‐face group programme (usual care) into a format suitable for email and videoconference delivery. At screening, participants provided written informed consent prior to commencing the study. Participants were asked to sign up to ground rules to facilitate effective group interaction and confidentiality, including accessing the video groups from a private area, free from disturbance. A test connection was made with each participant before joining the group programme to ensure audio and video connection. The programme was delivered to two groups between March and November 2019, using Skype for Business, by the first author, a registered Dietitian experienced in facilitating group weight management programmes. Participants accessed the groups from their own devices, usually from home, via the Internet. Participants used a variety of devices including desktop computers, laptops, tablets and smartphones.

The materials and content were based on an adaption of the in‐house NHS KindEating weight management group programme. The programme was based on NICE guidelines for lifestyle weight management services and delivered using a motivational interviewing communication style.^35^, ^36^ The programme provided eight weekly sessions followed by review sessions at 4 and 6 months. The interactive sessions covered different aspects of weight management including education on healthy eating and increasing physical activity, and employed the following behaviour change techniques: goal setting, problem solving, action planning, reviewing behaviour goals, self‐monitoring of behaviour, self‐monitoring of outcomes of behaviour, social support, instruction on how to perform behaviour, social comparison, behaviour substitution, habit formation, habit reversal, credible source and comparative imaging of future outcomes.^37^ Local service evaluation showed that conventional delivery of the programme led to an average weight loss of 4.42kg at 6 months. To adapt the programme for remote delivery, hands‐on activities were converted to visual activities. Interaction during remote delivery was predominantly limited to discussion, and small group discussion used in the traditional programme was replaced with whole group discussion. Written information and visual materials were sent by email the week before each session. Participants accessed reliable weighing scales from home and emailed their weekly weight records to the facilitator. Mean self‐reported weight loss for videoconference programme completers was comparable to the conventional programme.

### 2.3 Data collection and analysis

Participant experiences and perspectives of the study intervention were recorded in one‐to‐one videoconference or telephone semi‐structured interviews, conducted by the first author. Interviews took place 2 months from the start of the intervention, at the end of the first eight sessions. The topic guide was designed to investigate practical aspects of accessing the programme remotely, and topics relating to behaviour change facilitation were informed by SDT. Questions included the following: reasons for choosing this mode of access, advantages and disadvantages, ease of access, relationship with other group members and satisfaction with the programme. Interviews were transcribed verbatim, and Braun and Clarke^38^ thematic analysis was used to identify, categorize and describe participant experiences and perspectives of engaging with the programme via videoconference. A combination of inductive and deductive coding was used. Transcripts were initially analysed inductively to identify themes. In order to enhance understanding of how the intervention facilitated behaviour change, themes relating to behaviour change were deductively categorized within the theoretical framework of SDT.

## 3 RESULTS

**Fig. 2:**
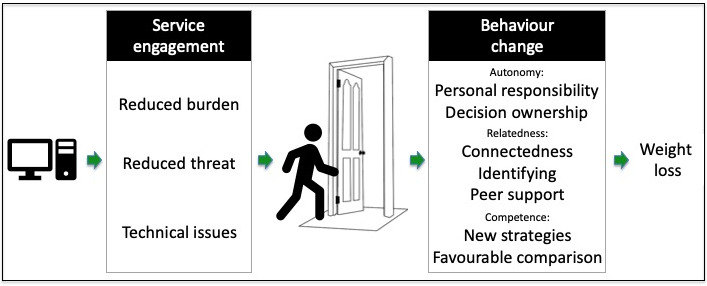
Themes in participant perspectives on accessing a weight management programme via videoconference

Participants’ *(*n = 13) experiences and perspectives of the weight management videoconference intervention are reported below with behaviour change facilitation themes described within the context of the SDT model of health behaviour change. Figure 2 illustrates the ten key themes as a process of service engagement to behaviour change facilitating weight loss. Participants have been assigned gendered pseudonyms.

### 3.1 Service engagement

The following themes relating to engagement were identified.

#### 3.1.1 Reduced burden

Participants felt that accessing the group via Skype was less burdensome compared to an in‐person group. It was important to many that it saved time, travel, monetary cost and employment disruption. For some participants, there were specific issues such as caring responsibilities and travel away from home that could be more easily accommodated by the flexibility in place of access that videoconference offered.

But, erm, it was really helpful because obviously you don’t have to go anywhere, you don’t have to waste petrol […]. This is less time because like, you just click on the thing. [Alys]

I wasn’t losing work and my boss wasn’t losing a man down either. [Elwyn]

…but obviously with being a carer for my wife…being at home it’s a lot easier… she does worry when I’m out for a period of time, but doing this on Skype, she’s sitting in the next room so if there’s something wrong she can give me a shout. [Howard]

I went to Harrogate the other week and I had to access from there on the iPad so…I would have missed the class if I’d been attending something. [Juliet]

When asked what they would have done if this option had not been available, some participants did not feel they would have been able to access any other support for weight management.

I don’t know, I’d still be out there looking most probably. That’s a difficult one. [Bob]

Probably would have continued to try and lose weight on my own, probably unsuccessfully…I had been referred before, but before the Skype sessions were an option and I couldn’t get to any of the in‐person sessions. [Glesni]

#### 3.1.2 Reduced threat

The remoteness of using videoconference had the advantage of feeling less daunting or stressful for people with more introverted personalities. It therefore seemed less threatening. Participants also benefitted from the added reassurance of being in familiar surroundings, in the comfort of their own homes, providing feelings of security. Participants also implied that the perceived confidentiality afforded by accessing a programme from their own home may be seen as a way of reducing the threat of exposure to the stigma and discrimination associated with obesity.

I think in a [meeting] room would be more intimidating, but I think on here it’s much more easier to, like, say how you’ve done this week and stuff. It’s easier to be honest and open on here than it is in a room full of people. [Alys]

…going to a group can be quite daunting and talking about your weight or like your diet or whatever…but it’s just a bit less stressful cos you’re in your own environment and you can feel comfortable. [Darcy]

It’s just that I could actually just go and have a couple of minutes out of camera shot if I wanted to… [Howard]

Nobody else is aware really that I’m doing it perhaps…It’s just the way I am. Probably it’s a benefit of doing it this way. It’s nice to just do it privately isn’t it. [Iris]

#### 3.1.3 Technical issues

In terms of feasibility, a key theme identified by participants was access to suitable equipment and adequate Internet access. In one case, unreliable connection caused by inadequate bandwidth resulted in the participant being unable to participate. Almost all participants reported some form of technical problems ranging from difficulty in installing the Skype for Business app, difficulty connecting to video or audio on their personal device, equipment incompatibility or unreliable Internet connection. The test video call was an important factor in overcoming initial difficulties. There was a common requirement for troubleshooting to ensure audio and video connection on each person's device. Although for others, connecting to the service was unproblematic.

I’ve just got a basic package [mobile phone contract] it does cut out now and again… I think maybe it’s cos I haven’t got a computer, maybe it would have been better if I’d got a computer. [Lynda ‐using smartphone]

First, I couldn’t see you and you couldn’t see me and then the, the volume. [Alys]

Oh, it’s pretty good, just go through your email and join the link. That's easy that is. [Bob]

Familiarity with some form of videoconferencing was a factor in openness to using this mode of communication. Those who chose to engage in this pilot study who may not have had prior experience, none‐the‐less generally considered videoconferencing to be normal, modern or commonplace. After initial connection, during access to the sessions, technical glitches and issues such as background noise, lost connection, videos freezing, images rotating and faulty links interfered to some extent with communication, but, although distracting, participants did not consider this to be a serious issue.

… as a medium I’m used to Skyping cos we use it nearly every day at work and it’s a lot easier than flippin’ travelling to go and see someone. [Mike]

…it’s just a bit more modern. You know, like, it’s good to talk to people on webcam and all that, like, it’s not anything out of the ordinary. [Darcy]

Because English is my second language, I need to pay more attention when other people are talking so, dropping and freezing and this type of thing were distracting, but not on an extent of spoiling the experience. It wasn’t a massive thing, you know, I could live with that. [Frank]

Well the con is that it drops out [the internet connection] it must have been twice, at least, each session I think…that was awkward…[Iris]

### 3.2 Behaviour change facilitation

In order to provide a clearer theoretical understanding of the role of this intervention in facilitation of behaviour change, themes relating to effectiveness were organized into the domains of autonomy, competence and relatedness, identified in SDT as leading to enhanced self‐motivation.

#### 3.2.1 Relatedness

Three themes relevant to relatedness were identified. Participants described how they felt connected to other group members, how they identified with other participants and felt supported by them.

##### Connectedness

Participants generally reported experiences suggesting they had been able to relate effectively to other participants via videoconferencing. With some suggesting they felt their relationships with others were actually enhanced by this mode of communication, compared to if they had attended an in‐person group.

It exceeded my expectations, let’s put it that way, because I didn’t think that seven or eight individuals would’ve been able to interact with each other as good as we did on the video link. [Juliet]

I think I meet them better like this than I would face‐to‐face […] if, you know, you’re in the room I wouldn’t be turning to like [name] and telling him stuff but when I’m facing him [on the screen] it’s better. [Elwyn]

##### Identifying with others

Participants generally felt they all had something in common. Several participants expressed the importance of there being someone else in the group with whom they identified, but they differed in how they categorized their peers for this purpose. Regardless of the remote communication, some participants related to each other on the basis of gender or age range, and others because of a similar health condition.

…but when you’ve got another male there as well it’s better, I think the conversation is different, same age group and working and things you know, so that’s it really. [Elwyn]

It was good to interact with them and to see their attitudes; the other people who were pre‐diabetic… [Iris]

##### Peer support

A common theme was the valuing of input and peer support or guidance from other group participants who shared the same challenges. Participants reported benefitting from practical ideas and suggestions based on the personal experiences of others.

It’s always good to just like pass stories or, you know, just give each other tips, like we’re all in the same boat, so it’s good to, like, bring each other along. [Darcy]

…and feel kind of supported in that, and like if I’m having a rough week I know that on Monday I can go and say ’I’ve found this difficult, does anybody else have this problem?’ or y’know ‘How do other people deal with this?’ [Glesni]

…knowing that other people in the same situation can get over those cravings, can change their habits, make it work. [Juliet]

#### 3.2.2 Autonomy

Themes of increasing personal responsibility and decision ownership were identified as relevant to autonomy.

##### Personal responsibility

Some participants started out hoping for an external solution (or even, in some cases, intervention from a higher power) but gradually took a more autonomous approach.

And all the time you’re searching for some magic that’s gonna happen. I think part way through I had the light bulb moment where I thought ‘It’s just you yourself, you’ve got to…. think, right, well, I’ve got to do this because nobody else can do it for you.’ [Juliet]

##### Decision ownership

Responses suggest that participants did not perceive behaviour changes were externally imposed, rather, they considered the options and internalized their decisions to inform real‐life behaviour changes. Participants demonstrated decision ownership by using first person language to describe how decisions were made to change their eating behaviours.

I decided what I thought I needed to change, that might work for me, based on what was discussed [Iris]

And so, I thought to myself ‘Right, well, what I have to do is, if I want potatoes, I don’t have bread’. [Juliet]

#### 3.2.3 Competence

The themes of learning new strategies and comparing oneself favourably with others were identified as influencing competence satisfaction amongst participants.

##### New strategies

Some participants reported learning new strategies as a result of the videoconference intervention. Visual aids, emailed information and discussion topics guided participant learning and development of practical skills including portion control and cooking skills.

I think this programme’s helped me realise, cos you know in the way that I am, like, using a smaller bowl instead of having a massive plate, it’s helped me realise that I don’t need all that food at once. [Darcy]

Well I thought like ‘Oh, I’m really rubbish at cooking so I’m not going to be able to do this, but then after realising, well you don’t need to be like an expert cook to be able to make simple easy changes’ [Alys]

##### Favourable comparison

Some participants perceived themselves as positive role models, more competent than their peers. Being able to give, as well as receive, useful tips, or being able to share one's expertise with others, emerged as a valuable means of boosting confidence.

…and I thought to myself ‘I’m a bit further down the road now’ […] It makes me feel better, that I can actually pass experience onto other people. […] I’m not the one sort of stuck at the back of the queue. [Howard]

…seeing how other people are struggling, then what I’m doing actually in comparison with how they were […]. That just gave me a good er, you know a good boost of morale for me. [Frank]

## 4 DISCUSSION

### 4.1 Service engagement

This study provides insights into the experiences of participants in engaging with a group weight management service via videoconference. Potential barriers to engagement with conventional group weight management programmes were described by participants. For these participants, the option of accessing a programme via videoconference sufficiently reduced the barriers to allow them to access support when they would otherwise have been unable, or chosen not, to do so.

Participants found that accessing the programme via videoconference reduced the burden on them in terms of travel, time, cost and employment disruption. These burdens associated with the requirement to travel to attend a centre can be particularly exaggerated in rural areas. Burden has been identified as an important component in acceptability of healthcare interventions.^39^ In a systematic review, intervention accessibility was identified as a facilitator in uptake of weight management programmes,^40^ and, in a feasibility study, McVay et al^41^ also identified practical factors as being important in the initiation of behavioural weight loss interventions. Women participating in a one‐to‐one postpartum weight management programme via video consultation in Australia similarly reported preferring the convenience and reduced logistical challenges of accessing a service from their own home.^28^

Reduced threat was also identified by participants as an important factor in facilitating access. Participants perceived that communicating with the group remotely was less daunting or intimidating than attending an in‐person group. They also perceived that accessing a programme from their own home afforded increased confidentiality, suggesting this may be seen as a way of reducing the threat of exposure to the stigma and discrimination known to contribute to avoidance of health care by people living with obesity.^42^ A report by Public Health England identified stigma as a barrier to uptake of group weight management services,^40^ and 15% of respondents in a USA survey (n = 3008) reported embarrassment as a barrier to seeking help.^43^ Thus, remote participation offers an important potential means of overcoming these barriers.

Almost all participants encountered some form of technical problems with the connection. In all but one case these problems were resolved by troubleshooting between the facilitator and participant before the first session, or they were not considered sufficiently significant to prevent participation. The multilevel mixed‐methods VOCAL study observed that technology‐related talk constituted 1%‐9% of the overall talk in virtual consultations. Examples included seeking reassurance such as ‘Can you see me?’, ‘I hope you can hear me OK?’^44^ In one study in Australia, 25% of all consultations experienced Internet connection dropping and sound issues.^45^ Thorough testing of equipment is recommended before providing a remote service to minimize any problems. Unexpected technical issues arising during implementation should be expected; therefore, strong information technology support is important.^46^

Familiarity with video calling was an important driver to openness to trying this mode of participation. The low proportion of potential participants choosing the videoconference intervention suggests that, when an in‐person alternative is available, the majority would prefer this. However, when there is no alternative option available, the uptake is likely to be higher.

### 4.2 Behaviour change facilitation

The results suggest that autonomy was supported by encouraging ownership of decisions regarding behaviour change goals. No evidence was found to suggest that delivering the programme remotely thwarted the support for autonomy. Indeed, the findings mirror those from a qualitative evaluation of a face‐to‐face programme where participants reported that facilitator delivery style was important in supporting their choice of set goals.^47^

Though participants described learning new strategies to manage their eating behaviour, it is interesting to note that much of the perceived increase in competence reported by videoconference participants was as a result of interaction with other group members, rather than from information provided by the facilitator. An important factor was favourable comparison with others, so that finding one was more competent in a certain behaviour, especially having the opportunity to share suggestions with others, was a powerful means of promoting feelings of mastery of change. This contrasts with the findings of Battista et al, who found that in a face‐to‐face lifestyle intervention programme, participants reported that particular ‘tools’ provided by the facilitator were most important in promoting competence.^47^ It is feasible that the adaption of hands‐on activities to visual activities and discussion reduced the memorability of practical aspects of the remotely delivered programme.

It has been established that groups can act to support weight management intervention delivery, and therefore behaviour change, but to realize this potential, participants need to experience a meaningful sense of social connection to other participants within the treatment group, so that they experience a shared social identity.^48^ A recent systematic review of qualitative evidence on service users' perspectives in weight management programmes found that service users were emphatic that supportive relationships, with service providers or weight management programme peers, are the most critical aspect of weight management programmes.^49^

Participants are more likely to adopt behaviours and values promoted by those with whom they feel connected.^30^ The need to feel a sense of belonging is important in fostering internalization, and the need to feel understood impacts on openness to information. Fostering participant relatedness in lifestyle intervention programmes is also important in increasing the likelihood of behaviour change maintenance.^47^ Discovering whether or not people were able to feel this sense of connection with other group members, and thus benefit from the peer support afforded by a group programme, even when they were not physically in the same place, was an important aspiration for this research.

Participants in this study generally reported that they had been able to relate effectively to other participants, with some expressing a preference for interacting in this way, perceiving that videoconference afforded the opportunity for more honesty and openness. One participant highlighted the benefit of ‘facing’ other group members on the screen, rather than sitting beside them in a physical meeting in fostering better relationships. There are limitations in using this mode; for instance, it is not possible for members to have casual one‐to‐one conversations with other group members with whom they identify more strongly. However, despite the limitations, participants generally felt they all had something in common and were ‘in the same boat’. They found other group members with whom they identified, and felt they were able to be supported, share ideas and learn from each other.

### 4.3 Implications for future research and practice

This research has taken on new relevance as requirements for social distancing during the COVID‐19 pandemic have focussed attention on new models of care that avoid face‐to‐face contact.^50^ It has been proposed that offering this alternative mode of access may help to reduce health inequalities related to rurality.^12^ However, there is a concern that in harnessing digital technology to overcome existing inequalities in access to health care, we might, inadvertently, increase inequalities of a different nature by excluding those who lack access to the Internet. The National Survey for Wales^51^ reported that 85% of households in Wales have access to the Internet including 78% of households in the most deprived areas. Thus, levels of digital exclusion remain. Barriers to digital inclusion include lack of digital skills and lack of access, including affordability and broadband connectivity.^52^ Internet access is lower amongst older adults, those living in more deprived areas, and those with poorer health.^13^

This study included a range of participant demographics, including a wide age range willing and able to access services digitally. However, none of the participants lived in an area in the most deprived quintile according to the Welsh Index of Multiple Deprivation.^53^ As over half the participants were classified as living in a rural area, it is worth noting that existing indices of deprivation have been criticized as being less relevant in capturing the nature of rural deprivation.^54^, ^55^ All of the participants were white, limiting the generalizability of the findings to other ethnic groups. The results suggest that videoconference groups are equally acceptable to men and women. This is an important consideration as engagement of men in conventional weight management programmes is low.^56^ Running groups remotely would allow the potential for groups to be convened on the basis of gender, language preference or condition rather than geographical location.

### 4.4 Strengths and limitations

The first author delivered the programme and conducted participant experience interviews. This approach had the advantage of allowing the researcher to build rapport with participants and thus may have facilitated the collection of richer qualitative data. Conversely, this approach may have introduced bias as participants may have been less inclined to report negative experiences or appear critical.

A major limitation of the study was that it did not capture the views of potential participants who did not respond to the invitation to access the programme, or those who chose to access the service via in‐person groups, as only those consenting to participate in the programme via videoconference were interviewed. Further research should explore the perspectives of those who chose not to engage with videoconference groups.

## 5 CONCLUSIONS

Delivering a weight management group programme via videoconference is feasible and may be the preferred option for some participants. Data from our small sample suggested that participants can still benefit from peer support despite not being physically in the same place. Offering this mode of access as an option may assist potential participants to overcome some of the barriers to access to standard in‐person programmes, particularly in rural areas. Strong information technology support will be required if group videoconference services are to become routine practice in the NHS. Further research should evaluate the effect of offering videoconference services on health inequalities and investigate if offering this alternative access option increases overall engagement. During the COVID‐19 pandemic, weight management programmes could utilize videoconference groups to continue to provide support.

## CONFLICT OF INTEREST

The authors declare no conflict of interest regarding this research.

## Data Availability

Data available from the authors upon reasonable request.
